# Predominance of CD4+ T cells in metastatic cervical lymph nodes in papillary thyroid carcinoma

**DOI:** 10.1530/EC-24-0135

**Published:** 2024-07-17

**Authors:** Camila Aparecida Moma, Icléia Siqueira Barreto, Ligia Vera Montali Assumpção, Denise Engelbrecht Zantut-Wittmann

**Affiliations:** 1 Endocrinology Division, Department of Internal Medicine, Faculty of Medical Sciences, University of Campinas, Campinas, São Paulo, Brazil; 2 Department of Pathology, Faculty of Medical Sciences, University of Campinas, Campinas, São Paulo, Brazil; 3 Endocrinology Division, Department of Internal Medicine, Faculty of Medical Sciences, University of Campinas, Campinas, São Paulo, Brazil

**Keywords:** CD4+ T lymphocyte, lymph node metastasis, lymphocytic subpopulation, papillary thyroid carcinoma

## Abstract

**Background:**

Papillary thyroid carcinoma has become increasingly prevalent over the years. Avoiding unnecessary treatments and the risk of complications is essential, as well as understanding the mechanisms of tumor progression and the conditions that indicate a worse prognosis. Assessment of the tumor microenvironment can allow us understand how the immune system organizes itself to contain neoplastic progression.

**Methods:**

We compared characteristics related to the lymphocytic subpopulations in the thyroid tumor microenvironment and lymph nodes in two groups, with and without lymph node metastatic involvement.

**Results:**

Of the 400 cases followed up at a thyroid cancer reference service, 32 were selected, of which, 13 cases did not present lymph node metastasis (N0 group) and 19 had lymph node involvement (N1 group). Clinical data were collected, and immunohistochemical reactions were performed for markers CD4, CD8, FoxP3, CD25, and CD20 in lymph nodes and peritumoral infiltrate. We found that the N1 group had larger tumor sizes, higher risk staging, higher frequency of extrathyroidal extension, shorter disease-free times, and higher expression of CD4+ T lymphocytes in lymph nodes; however, there was no difference in the expression of other markers or in the pattern of lymphocyte distribution in the lymph node.

**Conclusion:**

In cervical lymph nodes, the higher frequency of CD4+ T lymphocytes is related to the presence of metastasis. However, there were no differences in lymphocytic subpopulations in the thyroid tumor microenvironment. The absence of changes in unaffected lymph nodes could not predict any tumor behavior.

## Introduction

Thyroid cancer is the most common endocrine neoplasm, corresponding to 3.4% of all malignant neoplasms diagnosed annually. About 90% are differentiated tumors derived from thyroid follicular cells (1, 2, 3).

In recent decades, there has been an increase in the prevalence of thyroid cancer, mainly caused by papillary carcinoma, without a significant change in mortality (4). This increase in annual rate is believed to be due to the widespread use of diagnostic imaging methods and environmental risk factors such as obesity (3, 4, 5, 6).

Papillary thyroid carcinoma can occur multifocally, with about 30% corresponding to microcarcinomas (tumor diameter ≤ 1 cm), and most metastases arise in regional lymph nodes (7). The main driver mutations appear in the BRAF and RAS genes, leading to deregulation in MAPK pathway signaling, resulting in distinct clinicopathological characteristics and different gene expression and DNA methylation profiles (8).

According to the 5th edition of the WHO tumor classification, there are several subtypes of papillary carcinoma, with the tall cell subtype being the most common among the most aggressive. More than 90% of patients will survive for 20 years; however, factors such as male sex, advanced age, tumor size, extrathyroidal extension of the neoplasm, and incomplete surgical resection can negatively affect the prognosis (9).

An important aspect of understanding neoplastic aggressiveness is the assessment of the tumor microenvironment, which allows us to understand how the immune system orchestrates itself to contain neoplastic progression (10, 11, 12, 13, 14).

Initially, neoplastic cells are identified and destroyed by the immune system; however, with the accumulation of mutations, there is less tumor recognition, greater tumor resistance, and the development of an immunosuppressive environment, allowing the growth and expansion of cancer (15, 16, 17).

The tumor presents antigens, which are processed by antigen-presenting cells (such as dendritic cells, macrophages, or B cells). To trigger the activation of a T cell, antigen presentation through the major histocompatibility complex (MHC) and binding to a co-stimulatory protein are required (18).

When signals from MHC class I and costimulatory proteins occur, it is possible to activate CD8+ T lymphocytes, leading to apoptosis of tumor cells. However, when MHC class II signaling occurs, Th1 (hypersensitivity), Th2 (antibody production), Th17 (inflammation), or T reg (immunosuppression) activation may occur (17, 18).

Once Th17 is activated, IL-17 stimulation results in the production of cytokines that recruit more effector cells, such as natural killer (NK) cells and CD8+ T lymphocytes, leading to the death of the neoplastic cell. However, for effector cells, such as CD8+ T lymphocytes, to induce death, the tumor must express the protein on its surface via MHC I. Over time, tumors lose this expression, preventing the action of CD8+ T cells and, consequently, allowing evasion of the immune system.

As tumor cells no longer express MHC class I, the way to induce cell death is through NK binding to its receptor (killer-activating receptor – KAR); nevertheless, to sabotage the immune system, tumor cells reduce the expression of cytokines that attract NK cells, such as CXC ligand (16).

Another way to induce cell death is through the production of immunoglobulin G antibodies by B cells. Previous studies suggest that the presence of B cells correlates negatively with tumor size (19) and the expression of BRAF^V600E^ (20). However, they can also exert a pro-tumor function by secreting IL-10, which inhibits CD4+ and CD8+ T cells (21).

Camouflage is another immune system evasion mechanism, aiming to create an immunosuppressive environment through the recruitment of peripheral T reg cells, production of immunosuppressive enzymes and cytokines (such as IDO1, arginase, IL-10, and TGF-β), and expression of negative checkpoints (such as PDL-1/2) by the tumor (18, 22).

Therefore, there are different ways for the tumor to resist the immune system, and the more adaptation mechanisms it has, the greater the chance of neoplastic dissemination.

Previous studies showed that a greater presence of regulatory T lymphocytes (FOXP3+) and double-negative T lymphocytes (T CD3+ CD4− CD8−) was associated with papillary carcinoma compared to Hashimoto's thyroiditis (23). Likewise, a lower risk of PTC remission was observed in patients with a lower ratio of CD8+ / FOXP3+ T lymphocytes in chronic lymphocytic thyroiditis (14).

However, it is not well established in the literature whether the tumor and lymph node microenvironments are similar or not. Deliberating on the hypothesis that the environments respond differently, understanding how this occurs can have an impact on prognosis stratification and, consequently, on possible therapeutic targets for treatment.

Given this information, we aim to characterize the lymphocytic subpopulations present in both the thyroid microenvironment and lymph nodes, and thus evaluate possible differences in the presence or absence of lymph node metastasis.

## Methods

This is a retrospective cohort study in which, from a bank containing 1200 patients, 400 cases were preselected. All of them aged > 18 years old at diagnosis, with papillary thyroid carcinoma after total thyroidectomy and in follow-up for at least 12 months after surgery. Thirty-two cases met all the inclusion criteria, had complete data regarding clinical follow-up (especially about treatment, thyroglobulin levels, and cervical ultrasonography and scintigraphy), and had anatomopathological material available and suitable for immunohistochemistry (it should be well-preserved and have enough extension for the new sections, since the main objective was not to extinguish the structure). An additional exclusion criterion was the diagnosis of non-invasive follicular thyroid neoplasia with papillary-like nuclear features) (NIFTP); therefore, all the cases of subtype follicular papillary carcinoma were evaluated.

**Figure 1:**
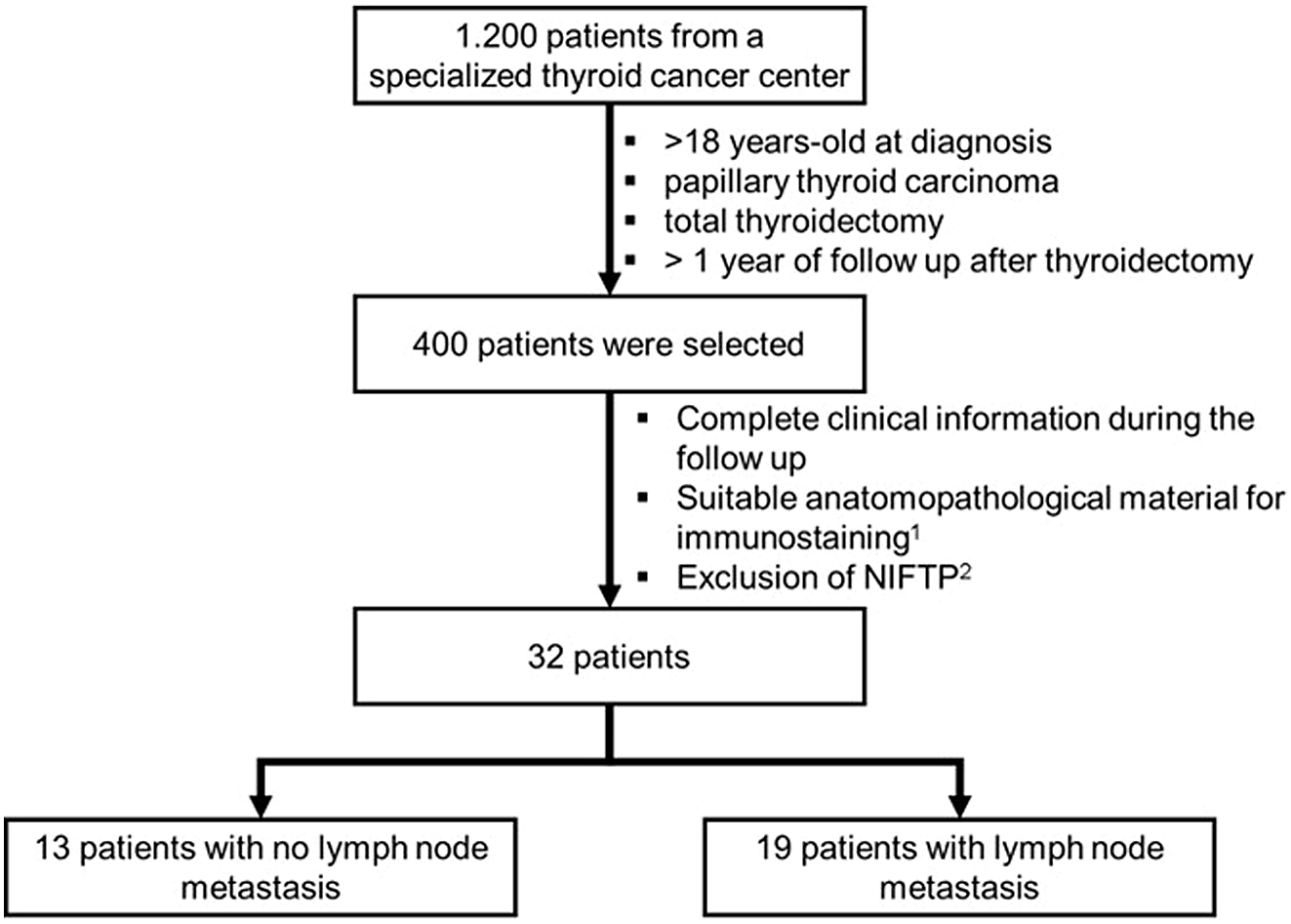
Patient selection for the study. 1. The anatomopathological material was considered suitable for immunostaining when it would not be extinguished after the sections (the main reason was not to completely lose the structures). 2. All cases with a previous diagnosis of papillary thyroid carcinoma, subtype follicular, were re-evaluated to exclude characteristics belonging to the noninvasive follicular thyroid neoplasm with papillary-like nuclear features (NIFTP).

The 32 selected cases were divided into two groups, one without lymph node involvement (N0 group), containing 13 patients, and another with lymph node involvement (N1 group), with 19 patients (Fig. 1). Information was collected from medical reports regarding patient and tumor characteristics, treatment, and follow-up. For histology and immunostaining, we evaluated both the thyroid tumor microenvironment and lymph nodes.

Regarding tumor features, PTC subtypes were described and later classified into ‘aggressive subtype’ (including tall cells and diffuse sclerosing ones) and ‘others’ (classic form and follicular subtype). All cases were staged by TNM staging and by the American Thyroid Association initial risk stratification (9).

Data were collected regarding radioiodine therapy, which was divided into ablative and adjuvant. The first one represents the administered activity after thyroidectomy for all patients, regardless of their TNM stage or ATA risk stratification. This was the medical protocol followed by the service until 2019; the standard activity was 100 mCi. However, depending on the associated factors, a higher activity could be an option for ablative therapy. The adjuvant radioiodine therapy was related to the total administered activity after, at least, 1 year of follow-up for those patients with an incomplete response.

We also collect data related to follow-up, such as disease-free time (number of months in which the patient did not present any evidence of biochemical and structural disease), total follow-up time (in months), and response to treatment (classified as excellent when there was no evidence of disease, and incomplete when there was still evidence of biochemical and/or structural disease). The incomplete biochemical response was defined as suppressed thyroglobulin (Tg) levels > 1 ng/mL, TSH-stimulated Tg > 10 ng/mL or rising anti-Tg antibody levels in the absence of structural disease (evaluated during the follow-up by whole body scintigraphy after radioiodine therapy and cervical ultrasonography with fine-needle aspiration biopsy).

We classified the type of infiltrate around the tumor according to its proportion in relation to the totality of the cells: minimal (< 5% of the area around the tumor), moderate (5–49%), and intense (≥ 50%).

For the N0 group, the largest lymph nodes were preferred, while for the N1 group, we chose those with some metastatic involvement. We describe the distribution pattern of lymphocytes in the lymph nodes, divided into follicular hyperplasia, paracortical hyperplasia, and sinus histiocytosis.

Sections stained with hematoxylin and eosin were evaluated for each case, and a representative paraffin tissue block was selected for immunohistochemistry studies. The sections were deparaffinized and rehydrated in an increasing gradient of alcohols. From this stage of the procedure, slides containing material collected for cytology were treated similarly to paraffin-embedded material. Antigenic retrieval was performed in 10 mM citrate buffer, pH 6, in a pressure cooker for 10 min, reaching room temperature. Endogenous peroxidase was blocked with 0.3% H_2_O_2_, and nonspecific binding sites were blocked with 3% BSA. The tissue was incubated overnight with anti-FOXP3 antibodies (rabbit C-terminal polyclonal IgG antibody clone E18492, SpringBio^®^), anti-CD25 (mouse monoclonal antibody clone 4C9, Cell Marque^®^), anti-CD4 (rabbit monoclonal antibody clone E204, Cell Marque^®^), anti-CD20 (mouse monoclonal antibody clone L26, Cell Marque^®^), and anti-CD8 (rabbit monoclonal antibody clone SP16, Cell Marque^®^) at 4ºC. The negative control was performed by replacing the antibodies with saline. The reaction was visualized using a streptavidin-biotin-immunoperoxidase system (Dako) with diaminobenzidine as the chromogen. Counterstaining was performed with hematoxylin.

**Figure 2:**
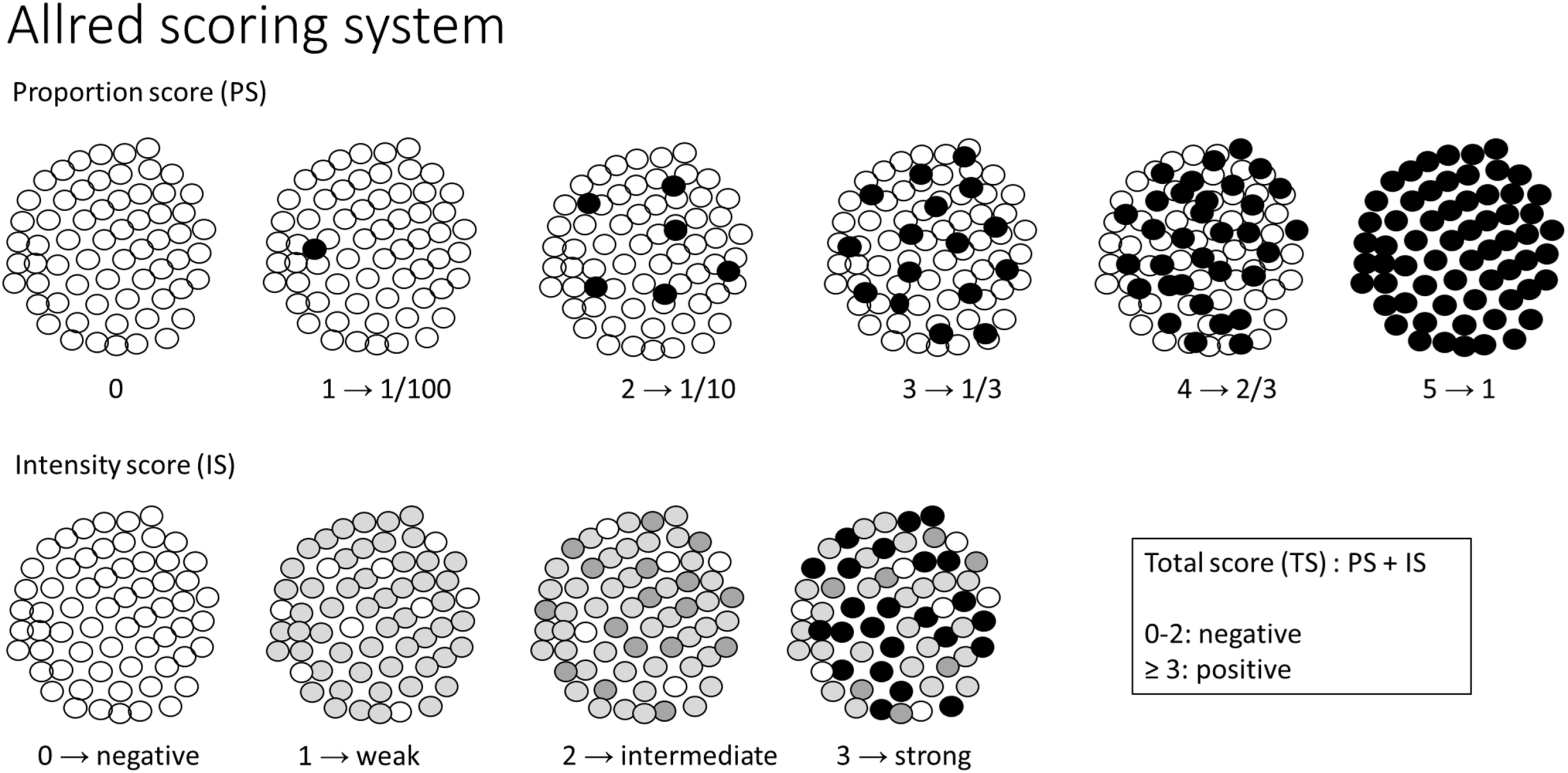
Representative diagram of the Allred scoring system. An unscaled image representing the Allred scoring system.

**Figure 3:**
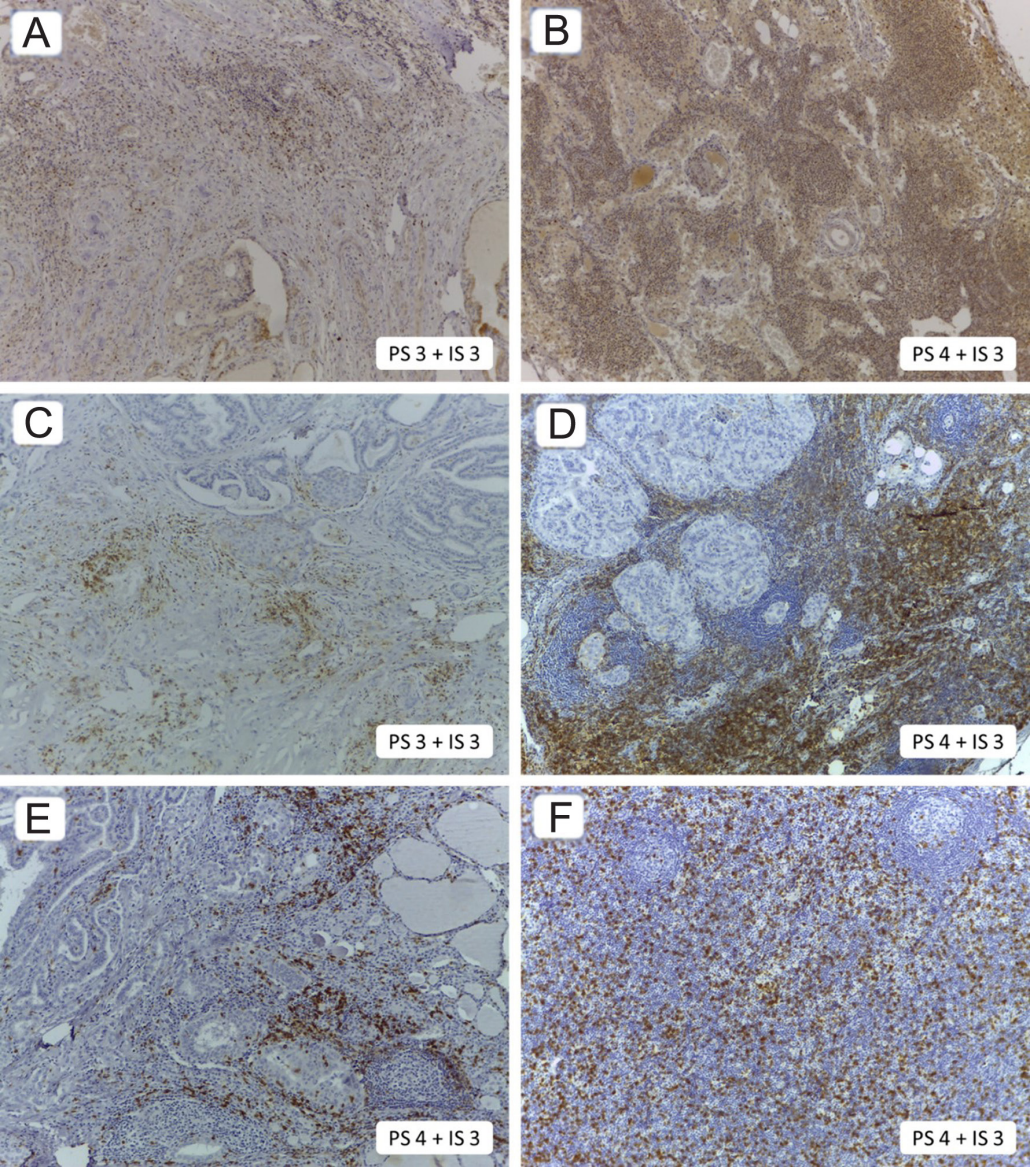
Examples of positive reactions for FOXP3, CD8, and CD4 antibodies. All the figures on the left show positive peritumoral infiltrates (A, C, and E) for FOXP3, CD4, and CD8 antibodies, respectively. All the figures on the right display positive lymph node lymphocytes (B, D, and F) for FOXP3, CD4, and CD8 antibodies, respectively. Magnitude view for A and C: 40×, for B, D, and E: 100×, and for F: 400×. Acquired images with Leica Microsystems software.

For the analysis of immunohistochemical reactions, the Allred scoring system (Fig. 2) was adopted (24), in which the number of immune cells reactive to the antibody (proportion score) and the intensity of the reaction in the cells (intensity score) were evaluated. The sum of the proportion and intensity scores generates an absolute value (numerical variable), which we also converted into a qualitative variable (negative if ≤ 2 or positive if ≥ 3). Below are representations of some positive reactions depicted in Fig. 3.

For statistical analysis, frequency tables were created for categorical variables, and measures of position and dispersion were calculated for continuous variables. The Chi-square test or Fisher's exact test was used to compare proportions as needed. For the comparison of continuous measures between two groups, the Mann–Whitney test was applied. For statistical tests, the significance level adopted was 5%.

Ethical approval for this study was obtained from the Ethics Committee in Research of the State University of Campinas (approval number 6.142.497). We confirm that written informed consent has been obtained from the involved patients, and they have given approval for this information to be published in this study.

## Results

### Assessment of characteristics of patients, tumor, treatment, and follow-up

Both the N0 and N1 groups did not differ in characteristics related to the patient, such as sex, skin color, age, smoking, and family history of papillary thyroid carcinoma, showing homogeneity between the groups (data not shown).

Concerning tumor characteristics, the N1 group had a larger tumor size, but there was no difference between groups in the frequency of microcarcinomas. The absence of extrathyroidal extension was associated with patients without lymph node metastases, while intermediate risk in the initial risk stratification was associated with patients who had lymph node metastases (Table 1).

**Table 1:** Comparison between groups N0 and N1 regarding tumor characteristics, treatment, and follow-up.

Characteristics			N0 (*n* = 13)	N1 (*n* = 19)	Total (*n* = 32)	*P*
PTC subtype		Aggressive	3 (23.08%)	10 (52.63%)	13 (40.62%)	0.1469^a^
		Others	10 (76.92%)	9 (47.37%)	19 (59.38%)	
Tumor extension		T1	8 (61.54%)	5 (26.32%)	13 (40.62%)	0.8500^a^
		T2	2 (15.38%)	3 (15.79%)	5 (15.62%)	
		T3	3 (23.08%)	2 (10.53%)	5 (15.62%)	
		T4	0 (0.00%)	9 (47.37%)	9 (28.12%)	
Distant metastasis		M0	11 (84.62%)	17 (89.47%)	28 (87.50%)	>0.999^a^
		M1	2 (15.38%)	2 (10.53%)	4 (12.50%)	
Focality		Unifocal	4 (30.77%)	8 (42.11%)	12 (37.50%)	0.7128^a^
		Multifocal	9 (69.23%)	11 (57.89%)	20 (62.50%)	
Tumor size		≤ 1 cm	3 (23.08%)	1 (5.26%)	4 (12.50%)	0.2788^a^
		> 1 cm	10 (76.92%)	18 (94.74%)	28 (87.50%)	
Tumor diameter (cm)			1.82 ± 1.24	2.81 ± 1.41	2.41 ± 1.41	**0.0283^b^**
Vascular invasion			3 (23.08%)	6 (31.58%)	9 (28.12%)	0.7036^a^
Extrathyroidal extension			2 (15.38%)	11 (57.89%)	13 (40.62%)	**0.0278^a^**
TNM stage		I	9 (69.23%)	14 (73.68%)	23 (71.88%)	0.6894^a^
		II	4 (30.77%)	4 (21.05%)	8 (25.00%)	
		III	0 (0.00%)	1 (5.26%)	1 (3.12%)	
ATA initial risk stratification		Low	6 (46.15%)	1 (5.26%)	7 (21.88%)	**0.0248^a^**
		Intermediate	5 (38.46%)	14 (73.68%)	19 (59.38%)	
		High	2 (15.38%)	4 (21.05%)	6 (18.75%)	
Ablative radioiodine dose (mCi)			130.30 ± 44.76	144.05 ± 26.04	139.31 ± 33.53	0.1210^a^
Adjuvant radioiodine dose (mCi)			–	166.58 ± 279.30	98.91 ± 228,48	–^c^
Disease-free time (months)			37.46 ± 44.29	17.11 ± 28.08	25.38 ± 36.34	**0.0344^a^**
Treatment response	Excellent		13 (100.00%)	12 (63.16%)	25 (78.12%)	–^c^
	Incomplete		0 (0.00%)	7 (36.84%)	7 (21.88%)	

Continuous variables are represented as the mean ± s.d., while categorical variables are expressed as frequency (*n* (%)). Bold indicates statistical significance.

^a^Fisher's exact test; ^b^Mann–Whitney test; ^c^Not enough observations to apply inferential testing.

Considering treatment characteristics, a shorter disease-free time was observed in the N1 group (Table 1).

### Assessment of peritumoral infiltrate and lymph node

Immunohistochemistry analyses were performed with categorical variables (negative, if a score from 0 to 2, and positive, if a score from 3 to 8 by the Allred scoring system) and with continuous variables to determine whether the score could correlate with the presence of lymph node metastasis. Around the tumor, there were no differences in the intensity of the peritumoral infiltrate or in the peritumoral lymphocyte subpopulations evaluated in this study (Table 2).

**Table 2:** Comparison between the N0 and N1 groups regarding the characteristics of the infiltrate around the tumor and its lymphocytic subpopulations in the thyroid tumor microenvironment.

Characteristic	N0 (*n* = 13)	N1 (*n* = 19)	Total (*n* = 32)	*P*
Infiltrate intensity				
Minimum	5 (38.46%)	5 (26.32%)	10 (31.25%)	
Moderate	3 (23.08%)	10 (52.63%)	13 (40.62%)	0.23362^a^
Intense	5 (38.46%)	4 (21.05%)	9 (28.12%)	
CD8				
Mean	6.15 ± 1.91	5.79 ± 2.62	5.94 ± 2.33	0.7387^b^
Positive	12 (92.31%)	16 (84.21%)	28 (87.50%)	0.62912^a^
CD4				
Mean	7.23 ± 0.83	6.89 ± 1.82	7.03 ± 1.49	0.8514^b,c^
Positive	13 (100.00%)	18 (94.74%)	31 (96.88%)	
CD25				
Mean	2.62 ± 2.02	3.42 ± 1.92	3.09 ± 1.97	0.3001^b^
Positive	8 (61.54%)	14 (73.68%)	22 (68.75%)	0.46661^d^
CD20				
Mean	5.15 ± 2.97	5.95 ± 2.17	5.62 ± 2.51	0.5423^b^
Positive	10 (76.92%)	17 (89.47%)	27 (84.38%)	0.37452^a^
FOXP3				
Mean	3.31 ± 2.06	3.11 ± 2.13	3.19 ± 2.07	0.9211^b^
Positive	10 (76.92%)	12 (63.16%)	22 (68.75%)	0.46732^a^
CD8/FOXP3 ratio in the peritumoral infiltrate	1.88 ± 1.08	2.21 ± 1.14		0.4044^b^

Continuous variables are represented as the mean ± s.d., based on their value in Allred scoring system, while categorical variables are expressed as frequency (*n* (%)). Number (percent) of positive cases for a specific antibody.

^a^Fisher's exact test; ^b^Mann–Whitney test; ^c^Not enough observations to apply inferential testing; ^d^Chi-square test.

### Assessment of the lymph node microenvironment

For the lymph node microenvironment, no distinction was observed in the pattern of lymphocytic distribution; however, there was a predominance of CD4+ T lymphocytes in the N1 group (Table 3). The analysis using categorical variables for all antibodies did not provide enough information to carry out the test; therefore, the data are not shown.

**Table 3:** Comparison between the N0 and N1 groups regarding histological characteristics and lymphocytic subpopulations in lymph nodes.

Characteristic	N0 (*n* = 13)	N1 (*n* = 19)	Total (*n* = 32)	*P*
T lymphocyte distribution				
Follicular hyperplasia	4 (30.77%)	7 (36.84%)	11 (34.38%)	0.3548^a^
Paracortical hyperplasia	3 (23.08%)	8 (42.11%)	11 (34.38%)	
Sinus histiocytosis	6 (46.15%)	4 (21.05%)	10 (31.25%)	
CD8	6.69 ± 0.63	7.11 ± 0.57	6.94 ± 0.62	0.06461^b^
CD4	6.31 ± 1.97	7.42 ± 0.77	6.97 ± 1.47	0.01231^b^
CD25	4.31 ± 1.18	4.68 ± 1.49	4.53 ± 1.37	0.49111^b^
CD20	5.92 ± 1.04	6.47 ± 0.77	6.25 ± 0.92	0.14891^b^
FOXP3	5.00 ± 0.82	4.53 ± 1.74	4.72 ± 1.44	0.50111^b^
CD8/FOXP3 ratio in the lymph node	1.46 ± 0.42	1.80 ± 0.75		0.1761^b^

Continuous variables are represented as the mean ± s.d., while categorical variables are expressed as frequency (*n* (%)).

^a^Fisher's exact test; ^b^Mann–Whitney test.

## Discussion

Understanding the tumor microenvironment is essential for individualizing PTC treatment, avoiding unnecessary interventions, and minimizing the risk of complications. Thus, we emphasize the importance of finding possible characteristics that distinguish, mainly within the lymph node microenvironment, the immune response to the aggressive effect of the tumor.

When analyzing the characteristics of the tumor, we noticed a larger diameter of the PTC and a greater occurrence of extrathyroidal extension in patients with metastases in cervical lymph nodes; however, there was no greater prevalence of aggressive subtypes. PTC size, histological type, extrathyroidal extension, and vascular invasion are associated with a worse prognosis (17). Consequently, as expected, some aspects related to tumor aggressiveness were more frequent in the N1 group.

The ablative activity received was similar between groups, as radioactive iodine therapy (RIT) was indicated as complementary therapy to thyroidectomy for carcinomas larger than 1 cm. However, there were no patients in the N0 group who required adjuvant therapy, a circumstance that did not allow a comparison with the N1 group.

Disease-free time was longer in the N0 group, probably due to the greater persistence of the disease and the need for complementary treatments in the N1 group, such as neck dissection and adjuvant RIT, for example. Due to the absence of patients with an incomplete response to treatment in the N0 group, it was not possible to perform a comparative analysis; however, we observed persistence/recurrence of the disease in more than a third of cases in the N1 group, inferring a worse response to treatment.

In the tumor microenvironment, there was no difference concerning the type of infiltrate around the tumor. Despite differences in our study design, Dvorkin and coworkers (11) demonstrated that the frequency of lymph node metastases and tumor size was lower in patients with PTC and Hashimoto's thyroiditis when compared to the group with PTC. Furthermore, patients were matched regarding disease staging, resulting in similar tumor size, demonstrating less need for RIT and a greater chance of being disease-free at the end of 1 year after thyroidectomy. Therefore, a higher occurrence of thyroiditis was expected in the group without evidence of lymph node metastases than in the group with proven metastatic lymph nodes, which we did not observe in our study.

However, Sakiz and coworkers (25), in a retrospective study, found no differences in tumor size and the presence of lymph node metastases between patients with PTC and chronic lymphocytic thyroiditis and patients with PTC only. Furthermore, they observed a higher frequency of multifocality and vascular, capsular, and neural invasion in the PTC group with chronic lymphocytic thyroiditis.

These contrasting data suggest that various factors could contribute to the different outcomes, such as environmental factors present in distinct populations. As a speculation, Xie (26) believes that the Epstein-Barr virus may be one of the contributors to genetic modulation and has the potential to influence the tumor microenvironment. Moreover, it is worth noting that the genetic profile of each neoplasm significantly influences the arrangement of immune cells, with the aim of camouflaging the tumor and sabotaging the anti-tumor immune system (16). Therefore, an ideal scenario would be to study the lymphocytic subpopulations of the tumor microenvironment, selecting papillary carcinomas based on the driver mutation.

In the lymph node microenvironment, we did not observe any distinction in the pattern of lymphocytic distribution; however, in the analysis of lymphocytic subtypes, there was a predominance of CD4+ T lymphocytes in patients with cervical lymph node metastasis. Thus, the greater expression of these CD4+ T lymphocytes was related to the presence of metastasis, and no other changes were found in the lymph nodes that could predict more aggressive tumor behavior.

In this study, we did not find a higher frequency of FoxP3+ cells in affected lymph nodes than in those without tumor invasion. In contrast, French and collaborators demonstrated that the presence of regulatory T lymphocytes (CD4+ FoxP3+) was greater in metastatic lymph nodes and was related to the aggressiveness of papillary carcinoma (27).

Consequently, we raised two hypotheses for the higher frequency of CD4+ T cells found in the affected lymph nodes. The first was that this higher number could be due to another subtype of T lymphocyte, such as CD4+IL17+ T cells (Th17). Studies in peripheral blood, with flow cytometry and IL-17 measurement, associated Th17 with malignant thyroid tumors; however, this lymphocytic subtype still has a controversial role, and little evidence shows an antitumor effect (28, 29, 30). Another point to be emphasized in the discussion about Th17 is that the cellular composition in peripheral blood does not always reflect the components of the tumor microenvironment, which could bring a completely different role for this T helper cell subtype, perhaps being one of the contributors to the expansion of the tumor (31).

On the other hand, the increase in the number of CD4+ T cells may reflect the greater activation of T lymphocytes in response to tumor invasion in the lymph node. In a previous study, the tumor microenvironment in the lymph node presented different characteristics from the microenvironment in the thyroid tissue, raising the hypothesis that the neoplasm escaped the immune system at the primary site; however, resistance was still found in the lymph node environment (32).

Another aspect evaluated was the ratio between CD8+/FoxP3+ T lymphocytes, which showed no difference between the groups. Infiltration by CD8+ T lymphocytes is associated with a better prognosis for differentiated thyroid carcinoma (19). However, in melanomas, the presence of CD8+ T lymphocytes associated with granzyme B positivity was greater in more advanced stages of the disease and, additionally, these cells were positively associated with the expression of PDL-1 and IDO in the tumor stroma (33). Recent data showed increased expression of PD-L1 in the PTC microenvironment despite the predominance of CD8+ T lymphocytes, which may be a possible mechanism to sabotage the immune system surveillance (22, 34). Such information shows us the need for a global understanding of the tumor microenvironment, considering the tumor, cell subtypes, and other components, such as cytokines, enzymes, and checkpoints.

A limitation of the study was the small number of patients included. Therefore, despite having a large database, the greatest difficulty was obtaining cases that met all inclusion criteria, with adequate material and a complete history. Another point was finding lymph nodes of adequate size for histological sections when there were no lymph node metastases. Although the groups were small, we observed that the inherent characteristics of the patients were homogeneous, reducing selection bias.

## Conclusion

In conclusion, the distribution pattern of lymphocytic subpopulations in lymph nodes showed a predominance of CD4+ T helper cells in patients with papillary thyroid carcinoma who had cervical lymph node metastasis, with similar characteristics observed in the lymph node microenvironment of patients with and without cervical lymph nodes metastases. There were no differences in lymphocytic subpopulations within the thyroid tumor microenvironment between patients with and without lymph node involvement. Furthermore, there were no changes in unaffected lymph nodes that could predict more aggressive tumor behavior. In the future, the evaluation of the genetic profile of papillary thyroid carcinomas and the interpretation of the immune response triggered in these tumor microenvironments, whether in the primary site or in the metastasis itself, may determine the best therapeutic regimen, in addition to estimating the patient's prognosis. More studies are needed to confirm the data found here.

## Declaration of interest

The authors declare that they have no conflicts of interest in carrying out this study that could have appeared to influence the work reported here.

## Funding

DEZ-W had a National Council of Technological and Scientific Development Scholarship (CNPq, grant number 303068/2021-3) and a research aid fund of the University of Campinas (FAEPEX number 2354/21).

## Availability of data and materials

All data generated or analyzed during this study are available from the corresponding author upon request.

## Author contribution statement

Conceptualization: CAM, DEZ-W; data curation: CAM, LVM; acquisition of resources: DEZ-W; investigation: CAM, ISB; methodology: CAM, DEZW; manuscript writing: CAM; manuscript review and editing: CAM, DEZ-W; supervision: DEZ-W.
